# Evaluating Surgical Approaches for Hemimandibular Hyperplasia Associated with Osteochondroma: A Systematic Literature Review

**DOI:** 10.3390/jcm13226988

**Published:** 2024-11-20

**Authors:** Luis Eduardo Almeida, Samuel Zammuto, Diego Fernando Lopez

**Affiliations:** 1 Surgical Sciences Department, School of Dentistry, Marquette University, Milwaukee, WI 53233, USA; 2 Orthodontics Department, Universidad del Valle, Cali 760001, Colombia

**Keywords:** hemimandibular hyperplasia, osteochondroma, temporomandibular joint disorders, condylectomy, orthognathic surgery, distraction osteogenesis, total joint replacement, genioplasty, facial asymmetry, mandibular function, temporomandibular joint surgery, maxillofacial abnormalities, 3D imaging techniques, recurrence rate, postoperative complications

## Abstract

**Background/Objectives**: Hemimandibular hyperplasia (HH) associated with osteochondroma presents complex challenges in maxillofacial surgery, including facial asymmetry, occlusal instability, and temporomandibular joint (TMJ) dysfunction. Surgical interventions vary widely in approach and outcomes, underscoring the need for a systematic evaluation of effectiveness. This systematic review assesses the effectiveness of surgical approaches for managing HH associated with osteochondroma, focusing on techniques including condylectomy, orthognathic surgery, distraction osteogenesis, total joint replacement (TJR), and genioplasty. **Methods**: Following PRISMA 2020 guidelines, a comprehensive search was conducted in PubMed, Scopus, and Web of Science for studies published from 2000 to 2023. Eligibility criteria were based on the PICO framework, with primary outcomes evaluated for facial symmetry, occlusal correction, mandibular function, and recurrence rates. The Cochrane Risk of Bias Tool assessed study quality, while the GRADE framework evaluated the certainty of evidence. This review was not registered due to exclusion criteria for certain dental topics in PROSPERO. **Results**: Of 145 studies identified, 18 met inclusion criteria, totaling 214 patients. High and low condylectomy both effectively corrected asymmetry, with high condylectomy reducing recurrence risk but often requiring reconstruction. Orthognathic surgery, combined with condylectomy, significantly enhanced facial symmetry and occlusal function. Distraction osteogenesis proved valuable for mandibular lengthening in cases of severe deformities, while TJR offered definitive solutions for extensive joint involvement. Genioplasty corrected chin asymmetry, contributing to improved facial balance. Limitations included small sample sizes and variable follow-up durations. **Conclusions**: Surgical approaches tailored to individual patient needs show effectiveness in treating HH associated with osteochondroma, achieving functional and esthetic outcomes. Future studies should prioritize larger cohorts and standardized follow-up protocols to better assess long-term efficacy. Advances in 3D surgical planning and individualized treatment strategies show promise for optimized patient-specific care.

## 1. Introduction

Hemimandibular hyperplasia (HH) is a rare, non-neoplastic condition characterized by asymmetric mandibular growth, leading to significant facial asymmetry, occlusal disturbances, and temporomandibular disorder (TMD). TMD affects approximately 34% of the global population, representing a substantial social and health concern due to its impact on functional impairment and quality of life [1]. First detailed by Obwegeser and Makek in 1986, HH is distinct from similar growth anomalies, such as hemimandibular elongation (HE), unilateral condylar hyperplasia (UCH), and condylar osteochondroma [2,3]. Typically manifesting during adolescence, HH progresses asymmetrically until skeletal maturity, resulting in esthetic deformities, occlusal changes, and masticatory difficulties [4,5].

Despite its low prevalence, HH presents clinical challenges due to its unpredictable growth patterns and variable presentation [6]. While the exact etiology remains unclear, HH is thought to be associated with hyperactivity of the condylar growth center. Advances in three-dimensional (3D) imaging and facial scanning have significantly improved diagnostic accuracy, enabling clinicians to differentiate HH from similar conditions and quantify asymmetry with greater precision [7,8]. López et al. (2019) highlighted the role of computed tomography (CT) and 3D reconstruction in assessing mandibular volumetric characteristics. This approach refines the classification of facial asymmetries like HH by analyzing parameters such as condylar length, mandibular ramus width, and symphysis deviation, thereby enhancing diagnostic and therapeutic decision-making [9].

Treatment modalities for HH primarily involve surgical management, with high condylectomy being a common intervention to halt aberrant condylar growth [10,11]. This procedure is often combined with orthognathic surgery to correct associated skeletal deformities, including occlusal canting and mandibular asymmetry [12,13]. However, there remains ongoing debate regarding the optimal timing and extent of surgical intervention, as well as the long-term outcomes of various approaches. While some studies advocate for early condylectomy to prevent further asymmetry, others suggest delaying surgery until growth stabilizes [14,15].

Several studies have examined outcomes associated with different surgical techniques for HH. For example, Sun et al. (2019) demonstrated that patients with HH frequently exhibit enlarged mandibular condyles and rami, which can be effectively assessed with 3D imaging modalities [3]. Walters et al. (2013) further emphasized the utility of automated asymmetry assessments to guide surgical planning and improve postoperative outcomes [4]. Despite these advancements, high-quality evidence comparing long-term functional and esthetic outcomes between various surgical approaches, such as condylectomy versus orthognathic surgery, remains limited.

This systematic literature review aims to critically evaluate surgical approaches in the treatment of HH associated with osteochondroma. By synthesizing data from relevant studies, we provide a comprehensive analysis of the effectiveness, outcomes, and potential complications of various surgical techniques, including condylectomy, orthognathic surgery, distraction osteogenesis, total joint replacement (TJR), and genioplasty. Additionally, this review examines recurrence rates of osteochondroma and the impact of these surgeries on facial symmetry, occlusal stability, and temporomandibular joint (TMJ) function. The findings contribute to the ongoing discussion regarding optimal treatment strategies for HH and aim to guide future clinical decision-making.

This review hypothesizes that specific surgical approaches, such as high condylectomy combined with orthognathic surgery, yield superior long-term outcomes in facial symmetry, occlusal stability, and TMJ function for patients with HH associated with osteochondroma. Tailored surgical interventions are anticipated to reduce recurrence rates, enhance functional and esthetic results, and improve patient quality of life compared to single-modality treatments.

## 2. Materials and Methods

This systematic literature review was conducted to evaluate the surgical approaches for treating hemimandibular hyperplasia (HH) associated with osteochondroma. The PRISMA 2020 statement served as the guideline for reporting, ensuring rigor and clarity in synthesizing findings [16].

### 2.1. Study Design

The primary objective of this review was to analyze the effectiveness and outcomes of surgical techniques, including condylectomy, orthognathic surgery (LeFort I osteotomy and sagittal split ramus osteotomy), distraction osteogenesis, total joint replacement (TJR), and genioplasty. This review strictly adhered to PRISMA 2020 guidelines to ensure replicability and consistency in study selection, data extraction, and analysis. The review was not registered in PROSPERO due to its exclusion criteria for dental and maxillofacial topics. However, adherence to PRISMA 2020 guidelines ensured rigor and transparency throughout the process.

### 2.2. Eligibility Criteria

Eligibility criteria were defined using the PICO (Population, Intervention, Comparison, Outcome) framework [17]:

Inclusion Criteria:

- Population: Human participants diagnosed with hemimandibular hyperplasia associated with osteochondroma.
- Intervention: Surgical management approaches, including condylectomy, orthognathic surgery, distraction osteogenesis, total joint replacement, or genioplasty.
- Comparison: Studies comparing different surgical techniques, or surgical versus non-surgical approaches.
- Outcome: Measurable outcomes related to facial symmetry, occlusal stability, mandibular function, and recurrence rates.

Exclusion Criteria:

- Population: Conditions unrelated to HH or temporomandibular disorders.
- Intervention: Non-surgical or experimental treatments outside the scope of this review.
- Outcome: Studies without specific outcome measures for facial symmetry, joint function, or recurrence.
- Study Type: Non-systematic reviews, case reports, opinion pieces, and studies not meeting systematic review standards.

This rigorous, evidence-based selection process enhanced the comprehensiveness and relevance of this systematic review.

### 2.3. Information Sources and Gray Literature Search

A comprehensive search was conducted across PubMed, Scopus, and Web of Science. PubMed was selected for its extensive biomedical literature coverage, Scopus for its interdisciplinary reach and inclusion of high-impact journals, and Web of Science for citation tracking and access to a broad spectrum of peer-reviewed journals. To reduce publication bias, gray literature searches included conference proceedings, government databases, and institutional repositories such as ClinicalTrials.gov and the OpenGrey database. The search covered studies from 2000 to 2023, capturing advancements in diagnostic imaging and surgical techniques. The final search was completed in October 2024, with keywords including “hemimandibular hyperplasia”, “osteochondroma”, “condylectomy”, “orthognathic surgery”, “distraction osteogenesis”, “total joint replacement”, and “genioplasty”. Reference lists of relevant articles were also reviewed to identify any studies missed in the database search.

### 2.4. Search Strategy

The search strategy employed Boolean operators, Medical Subject Heading (MeSH) terms, and free-text keywords to create a targeted query: (“hemimandibular hyperplasia” OR “osteochondroma”) AND (“condylectomy” OR “orthognathic surgery” OR “distraction osteogenesis” OR “total joint replacement” OR “genioplasty”), with filters for human studies and peer-reviewed publications applied.

### 2.5. Selection Process

**Figure 1:**
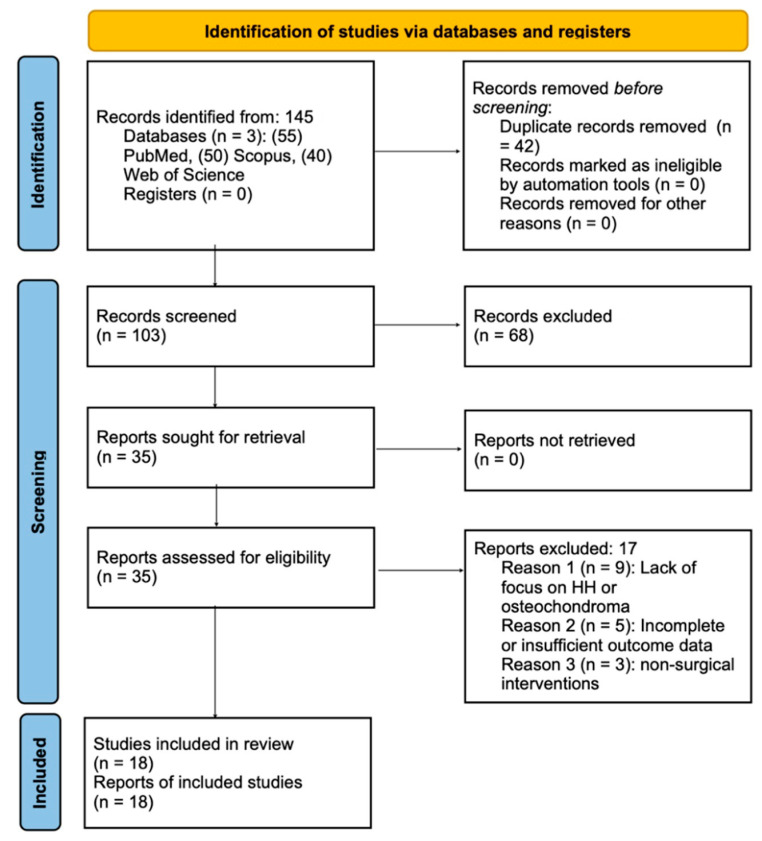
PRISMA 2020 flow diagram for the selection of studies evaluating surgical approaches for hemimandibular hyperplasia associated with osteochondroma.

Following the PRISMA flow diagram, 145 studies were initially identified. After removing 42 duplicates, 103 unique studies remained. Two independent reviewers screened titles and abstracts for relevance, retrieving 35 full-text articles for eligibility assessment. Eighteen studies met the criteria and were included in the final review. Discrepancies were resolved through discussion or consultation with a third reviewer, as illustrated in Figure 1, which clarifies the final study inclusion.

### 2.6. Data-Collection Process

Two reviewers independently extracted data using a standardized form. Data items included study design, sample size, type of surgical intervention, outcomes (e.g., facial symmetry, occlusal correction), follow-up duration, recurrence rates, and complications. Disagreements were resolved through discussion or by consulting a third reviewer.

### 2.7. Data Items

Primary data items collected were surgical intervention type, number of patients, follow-up duration, and key surgical outcomes (facial symmetry, occlusal correction, mandibular function, and recurrence rates). Secondary items included demographic details and postoperative complications.

### 2.8. Risk-of-Bias Assessment

The Cochrane Risk-of-Bias Tool was used to assess selection, performance, detection, and reporting bias. A minimum sample size of 15 patients was established to ensure statistical power and improve external validity. Studies with fewer than 15 participants or shorter follow-up periods were classified as moderate risk. Four studies were assessed as moderate risk due to smaller sample sizes or shorter follow-up, while 14 studies were categorized as low risk overall.

### 2.9. Synthesis Methods

A narrative synthesis was employed due to heterogeneity in study designs, surgical techniques, and outcomes, precluding meta-analysis. Studies were grouped by intervention, and key outcomes were summarized in tables for visual representation.

### 2.10. Reporting-Bias Assessment

Completeness of outcome reporting was evaluated to assess reporting bias, particularly for postoperative complications and recurrence rates. Studies with incomplete reporting were noted as having a higher risk for reporting bias.

### 2.11. Certainty Assessment

The GRADE framework was used to evaluate evidence certainty based on risk of bias, directness, consistency, and precision:

- Risk of Bias: Low in studies with clear inclusion criteria, comprehensive outcome reporting, and sufficient follow-up.
- Directness of Evidence: High, as all studies addressed relevant populations, interventions, and outcomes directly.
- Consistency of Results: Moderate, with minor inconsistencies in recurrence rates attributed to differences in follow-up duration.
- Precision of Effect Estimates: Limited in studies with smaller sample sizes, although larger studies provided more robust estimates.

The GRADE assessment indicated moderate-to-high confidence in findings, especially regarding high condylectomy combined with orthognathic surgery and TJR interventions.

### 2.12. Meta-Analysis Explanation

A meta-analysis was not feasible due to heterogeneity in study designs, surgical techniques, and outcome measures. The diverse interventions and variable follow-up durations across studies prevented data pooling, making narrative synthesis the most appropriate approach.

### 2.13. Authorship Roles and Conflict Resolution

The primary author led the study design, search strategy, and data extraction. The secondary author verified data and contributed to the analysis, while a third author oversaw bias assessment and synthesis. Conflicts were resolved through consensus, with consultation from a senior author if required to ensure consistency and reliability.

### 2.14. Excluded Studies

To ensure transparency, the following studies were excluded during the full-text review stage due to not meeting predefined inclusion criteria, such as a focus on non-surgical approaches, unrelated pathologies, or experimental models not applicable to HH and osteochondroma [18,19,20].

Additional excluded studies, along with detailed reasons for exclusion, are documented in Supplementary Materials to ensure a comprehensive and transparent review process.

## 3. Results

A comprehensive literature search was conducted across PubMed, Scopus, and Web of Science, yielding a total of 145 studies related to hemimandibular hyperplasia (HH) associated with osteochondroma. After removing 42 duplicate records, 103 unique studies remained for screening. Titles and abstracts of these studies were screened based on predefined inclusion criteria, resulting in the exclusion of 68 studies. This process led to the retrieval of 35 full-text articles for a thorough eligibility assessment.

Following the full-text review, 18 studies met the inclusion criteria and were incorporated into the systematic review. These studies provided detailed reports on surgical interventions for HH associated with osteochondroma, focusing specifically on outcomes such as facial symmetry, occlusal correction, and temporomandibular joint (TMJ) function.

The remaining 17 studies were excluded for the following reasons:

- Nine studies lacked a specific focus on hemimandibular hyperplasia or osteochondroma, instead addressing unrelated jaw conditions.
- Five studies had incomplete or insufficient outcome data, making reliable conclusions unattainable.
- Three studies focused on non-surgical interventions or experimental models, which fell outside the scope of this review.

The study-selection process is summarized in the PRISMA flow diagram (Figure 1) outlining the identification, screening, and final inclusion of studies.

### 3.1. Study Characteristics

This review includes 18 studies, with a combined total of 214 patients diagnosed with hemimandibular hyperplasia (HH) associated with osteochondroma. Sample sizes across the studies varied significantly, from single-patient case reports to larger prospective studies with up to 57 patients.

The study designs included:

- Seven case reports.
- Nine case series.
- Two prospective cohort studies.

The surgical interventions assessed across these studies comprised:

- Condylectomy (both high and low).
- Orthognathic surgery (LeFort I osteotomy and sagittal split ramus osteotomy).
- Distraction osteogenesis.
- Total joint replacement (TJR).
- Genioplasty.

Follow-up periods ranged from 6 to 36 months, with outcomes evaluated based on key indicators, including:

- Facial symmetry.
- Occlusal correction.
- Mandibular function.
- Recurrence of osteochondroma.

The following tables provide a comprehensive summary of each study’s main characteristics, detailing the surgical techniques used, outcomes assessed, follow-up durations, and sample sizes.

### 3.2. Risk of Bias in Studies

The risk of bias for the included studies was evaluated using the Cochrane Risk-of-Bias Tool, adapted for observational and case study designs. The assessment identified the following:

- Low Risk of Bias: Fourteen studies demonstrated a low risk of bias, characterized by clear inclusion criteria, comprehensive reporting of surgical outcomes, and adequate follow-up durations. These factors contribute to the reliability and consistency of the data presented.
- Moderate Risk of Bias: Four studies were classified as having a moderate risk of bias. These studies were limited by small sample sizes and shorter follow-up periods (less than 12 months), which restrict the reliability of long-term outcome data. Additionally, these studies provided less comprehensive information on postoperative complications, potentially affecting a full understanding of surgical risks.

Selection bias was generally well-controlled across the studies due to the use of transparent inclusion and exclusion criteria. However, reporting bias was observed in three studies, where incomplete reporting of postoperative complications limited the thoroughness of outcome analysis.

Most studies included in this review demonstrated strong methodological quality, offering valuable insights into the surgical management of hemimandibular hyperplasia associated with osteochondroma. Ensuring consistent postoperative follow-up and thorough complication reporting are critical steps to further minimize bias and enhance the robustness of future studies.

### 3.3. Results of Individual Surgical Techniques

#### 3.3.1. Condylectomy

Condylectomy is a primary surgical approach for managing hemimandibular hyperplasia (HH) associated with osteochondroma of the mandibular condyle, aiming to remove pathological condylar growth, restore functional occlusion, and achieve facial symmetry. The procedure can be performed in the following forms:

- Condylar Shaving: Removal of approximately 3 mm from the condylar head.
- High Condylectomy: Removal of about 5 mm from the condylar head, spanning the medial to lateral pole.
- Low Condylectomy: Removal exceeding 5 mm, typically determined by the degree of asymmetry.
- Proportional Condylectomy: Tailored to the specific asymmetry observed, where the amount removed is proportionate to the deformity [9,10].

Bone scintigraphy, particularly single photon emission computed tomography (SPECT), plays a vital role in distinguishing active condylar hyperplasia from passive HH phases. López et al. (2016) demonstrated that SPECT was more sensitive than planar bone scintigraphy for detecting condylar hyperactivity, identifying 52.46% of unilateral condylar hyperplasia (UCH) cases compared to only 13.11% with planar imaging, underscoring SPECT’s importance in assessing the need and extent of surgical intervention [15]. Additionally, Yu et al. (2019) found that osteochondroma cases showed greater radiopharmaceutical uptake than condylar hyperplasia, indicating higher proliferative activity and mineralization. This distinction is essential for precise surgical planning in osteochondroma cases [21].

The surgical approach used in condylectomy can impact the risk of facial nerve injury, which varies with incision technique. Common incisions include the preauricular, endaural, and modified endaural approaches, each offering specific benefits. The endaural approach allows substantial access to the temporomandibular joint (TMJ) while minimizing nerve injury risk. In a retrospective study, Pauwels et al. (2022) reported that when the endaural approach was performed with sharp dissection, facial nerve injury rates were low (1.17%), with only transient impairments primarily affecting the temporal branch [22]. Electromyographic studies further support the use of minimally invasive techniques to assess nerve function, particularly for patients with prior TMJ surgeries or prolonged operative times [22].

Additionally, Fariña et al. (2016) compared high versus proportional condylectomy in UCH patients, suggesting that proportional condylectomy may prevent the need for secondary orthognathic surgery. By customizing the excision based on asymmetry, proportional condylectomy provides a balanced approach that achieves both functional and aesthetic outcomes, potentially reducing the need for further surgical interventions [11].

##### High Condylectomy

High condylectomy, which involves the complete removal of the condylar head, is generally indicated in cases with extensive condylar involvement, such as significant osteochondroma, to ensure comprehensive resection of pathological growth. This approach reduces the likelihood of recurrence but often necessitates subsequent joint reconstruction to restore function and maintain stability.

Si et al. (2023) demonstrated the application of a 3D-printed cutting guide in high condylectomy, enhancing surgical precision and contributing to significant improvements in facial symmetry [23]. Their study, with a 12-month follow-up, reported no recurrence of osteochondroma, highlighting the potential of advanced technologies to optimize outcomes in condylar surgery [23].

In a case report by Park et al. (2015), high condylectomy was combined with bimaxillary orthognathic surgery to address severe facial asymmetry. The patient experienced substantial improvements in TMJ function and facial symmetry with no postoperative complications or recurrence, underscoring the effectiveness of this combined approach for complex asymmetry cases [24].

Morey–Mas et al. (2011) described a successful high condylectomy followed by immediate prosthetic reconstruction using a total stock prosthesis [25]. This approach provided stable joint function and occlusion, with no recurrence observed over a 24-month follow-up period, demonstrating that immediate prosthetic reconstruction can be an effective solution following extensive condylar resections [25].

Ghawsi et al. (2016) conducted a systematic review on high condylectomy for unilateral condylar hyperplasia (UCH), concluding that high condylectomy is an effective treatment option for cases requiring substantial condylar removal. However, the review noted variability in etiology, diagnostic methods, and timing of interventions across studies, indicating a need for standardized protocols to optimize functional and aesthetic outcomes [26].

While high condylectomy involves the complete removal of the condylar head, it is distinct from low condylectomy, which typically resects over 6 mm but retains part of the condylar structure. This differentiation is crucial for accurately categorizing the extent of resection and its potential impact on TMJ function.

##### Low Condylectomy

Low condylectomy is a conservative approach aimed at preserving much of the condylar structure by removing only the affected portion of the condyle. This method is preferred when osteochondroma is localized, as it reduces the need for joint reconstruction and supports faster recovery. Although it carries a slightly higher risk of incomplete resection and potential recurrence compared to high condylectomy, low condylectomy remains effective for cases requiring minimal resection.

Mamatha et al. (2015) reported a rare case of osteochondroma at the mandibular angle successfully treated with low condylectomy [27]. The patient exhibited an excellent recovery with no recurrence, illustrating the efficacy of this conservative approach for treating localized osteochondromas [27].

In a study by Kim et al. (2015) involving five patients with mandibular condyle osteochondroma, low condylectomy effectively restored facial symmetry and joint function across all cases. Follow-up periods ranging from 12 to 24 months showed no recurrence, highlighting the reliability of low condylectomy for managing localized mandibular lesions [28].

Wolford et al. (2014) conducted a retrospective review of 37 cases, demonstrating that low condylectomy combined with orthognathic surgery yielded excellent functional and aesthetic outcomes. This combined approach minimized complications and facilitated faster recovery, suggesting that low condylectomy can be effectively integrated with other surgical interventions to enhance overall outcomes [10].

Fariña et al. (2015) explored low condylectomy as a sole treatment for active unilateral condylar hyperplasia (UCH). Their observational study showed that low condylectomy alone significantly improved facial symmetry, occlusal alignment, and skeletal balance, particularly in cases of severe asymmetry. Using panoramic X-rays to align the condylar segment on the affected side with the healthy side, they observed marked improvements in chin deviation, occlusal plane tilt, and mandibular angle symmetry, establishing low condylectomy as an effective etiological treatment for UCH [29].

Low condylectomy typically involves resecting more than 6 mm of the condylar height from the affected area while retaining a substantial portion of the condylar head. This distinction is crucial for categorizing the surgical approach accurately and understanding its potential functional impact on the TMJ.

Additionally, Mehra et al. (2016) compared outcomes of complete condylectomy with joint replacement versus low condylectomy with joint preservation in cases of TMJ condylar osteochondroma. Their study highlighted that low condylectomy combined with joint preservation could be an effective alternative, reducing invasiveness while maintaining function, especially in cases where complete condylectomy may not be necessary [30].

##### Comparative Outcomes and Long-Term Stability

High and low condylectomy are both effective surgical approaches for managing hemimandibular hyperplasia (HH) associated with osteochondroma, each with distinct advantages. High condylectomy, involving the complete removal of the condylar head, allows for more extensive resection and reduces recurrence risk but often requires subsequent joint reconstruction. In contrast, low condylectomy preserves a greater portion of the condylar structure, facilitating faster recovery and reducing the need for reconstruction, though it carries a slightly higher risk of incomplete resection and potential recurrence.

Mehra et al. (2016) compared outcomes between low condylectomy with joint preservation and complete condylectomy with joint replacement, finding that both techniques achieved similar functional outcomes. However, low condylectomy allowed for faster recovery and preserved joint function, providing an effective alternative to more radical resection [30].

Qi et al. (2021) highlighted the use of advanced resection guides, especially in high condylectomy, to enhance precision in condylar resection, reduce recurrence risk, and improve surgical outcomes. Their study demonstrated that precise resection guides are instrumental in ensuring complete tumor removal and maintaining long-term stability [23,31].

Lu et al. (2020) assessed long-term stability in patients undergoing orthognathic surgery combined with condylectomy. Their findings indicated that both high and low condylectomy provided stable outcomes over extended follow-up periods, with no significant relapse or recurrence, supporting the effectiveness of both approaches in maintaining facial symmetry and joint function [32] (Table 1).

In summary, condylectomy, whether high or low, is an essential surgical technique in managing HH associated with osteochondroma, offering significant improvements in facial symmetry, occlusion, and joint function. High condylectomy provides comprehensive resection by removing the entire condylar head, which reduces the risk of recurrence but often requires joint reconstruction. Low condylectomy, by preserving a larger portion of the condyle, promotes faster recovery and reduces reconstruction needs, albeit with a slightly higher recurrence risk due to incomplete resection.

The choice between high and low condylectomy should be individualized, considering the extent of condylar involvement, the patient’s functional and aesthetic goals, and the surgeon’s objective of balancing recovery with long-term stability. Advances in surgical technology, such as 3D-printed cutting guides, have enhanced the precision of both procedures, contributing to more predictable and stable outcomes. Long-term follow-up remains essential to monitor for recurrence or complications, ensuring the durability of surgical results.

#### 3.3.2. Orthognathic Surgery (LeFort I Osteotomy and SSRO)

Orthognathic surgery plays a crucial role in addressing the functional and esthetic deformities caused by hemimandibular hyperplasia (HH) associated with osteochondroma. By combining orthognathic procedures with condylectomy, surgeons can restore facial symmetry, correct occlusion, and enhance overall jaw function. Recent advances in 3D surgical planning, imaging, and personalized surgical guides have further improved outcomes in these complex cases.

##### Surgical Techniques and Outcomes

Orthognathic surgery, particularly when combined with condylectomy, has shown consistent success in enhancing facial symmetry and occlusal alignment for patients with HH and osteochondroma. Park et al. (2015) documented a case of a 39-year-old female who underwent bimaxillary orthognathic surgery along with condylectomy, resulting in substantial improvements in TMJ function and facial symmetry, with no recurrence observed after a 12-month follow-up [24].

Wilson et al. (2015) reported on a staged approach involving an initial condylectomy followed by orthognathic surgery, which produced stable aesthetic and functional results over a 24-month follow-up period [33]. Similarly, Lim et al. (2014) described the outcomes of simultaneous orthognathic surgery and tumor excision in a patient with HH, achieving satisfactory facial symmetry and occlusal alignment without recurrence over a 3-year follow-up period [34].

Fariña et al. (2016) conducted a comparative study of high condylectomy and proportional condylectomy when combined with orthognathic surgery [11]. In cases requiring high condylectomy, an average of 5 mm was removed, while proportional condylectomy required an average resection of 9 mm to balance the affected and unaffected sides [11]. Proportional condylectomy significantly reduced the need for additional orthognathic surgeries, achieving effective functional and esthetic outcomes with fewer secondary procedures (*p* < 0.001), making it a viable standalone treatment for unilateral condylar hyperplasia (UCH) [11].

Recent advancements, such as 3D surgical guides, have further refined these combined surgical approaches. Si et al. (2023) demonstrated the utility of 3D guides in enhancing precision during tumor excision and optimizing orthognathic alignment. The study highlighted that 3D guides contribute to more accurate resection and improved esthetic outcomes, supporting stable, long-term results for patients with complex mandibular asymmetries [23].

##### Complications

Orthognathic surgery yields substantial improvements in facial symmetry and jaw function for patients with hemimandibular hyperplasia (HH) associated with osteochondroma, but it is not without potential complications. Common complications include infection and occlusal relapse. Wan et al. (2019) reported that while most patients experienced favorable outcomes, a minority encountered occlusal relapse and postoperative infections, suggesting the need for comprehensive postoperative care [35]. Similarly, Wu et al. (2018) observed a risk of occlusal relapse in long-term follow-ups, underscoring the importance of careful monitoring and the use of retainers or other corrective measures to maintain occlusal stability [36].

##### LeFort I Osteotomy in the Treatment of Hemimandibular Hyperplasia Associated with Osteochondroma

LeFort I osteotomy is a foundational technique for correcting maxillary deformities and asymmetries associated with hemimandibular hyperplasia (HH) and osteochondroma. This procedure addresses both functional and esthetic concerns by repositioning the maxilla to restore occlusal balance and facial harmony. LeFort I osteotomy is frequently performed in combination with other procedures, such as condylectomy or sagittal split ramus osteotomy (SSRO), to achieve comprehensive correction of facial asymmetry and optimize occlusal alignment. These combined approaches allow for a tailored treatment plan that addresses the complex presentation of HH, supporting stable and aesthetically pleasing outcomes for patients with severe maxillofacial deformities.

##### Indications for LeFort I Osteotomy

LeFort I osteotomy is indicated for patients with significant facial asymmetry and occlusal plane canting caused by hemimandibular hyperplasia (HH) or osteochondroma. This procedure is especially beneficial when maxillary deformity contributes to malocclusion or functional disturbances, as it effectively repositions the maxilla to achieve occlusal balance and facial symmetry. Occlusal plane canting in these patients often stems from a vertical skeletal discrepancy, where the alveolar process on the hyperplastic side shows greater development than the unaffected side, resulting in an inclined maxillary plane.

Huang et al. (2024) examined the use of LeFort I osteotomy in combination with sagittal split ramus osteotomy (SSRO) and simultaneous total joint prosthesis (TJP) reconstruction in patients with unilateral temporomandibular joint (TMJ) ankylosis and associated jaw deformity [37]. This comprehensive approach allowed for precise correction of asymmetry and occlusal alignment, addressing both functional and esthetic concerns. By incorporating TJP, the study demonstrated an effective method for preventing ankylosis recurrence, yielding substantial improvements in facial symmetry and joint function. This underscores the value of combining osteotomy with advanced joint reconstruction techniques to enhance long-term stability in complex cases [37].

Additionally, Seres et al. (2014) reported on a patient who underwent LeFort I osteotomy using computer-aided planning and 3D rapid prototyping. This method facilitated precise surgical planning and resulted in significant improvements in facial symmetry and functional stability, showcasing the advantages of modern technology in achieving optimized surgical outcomes [38].

##### Techniques and Modifications

The LeFort I osteotomy involves a horizontal osteotomy across the maxilla, allowing for three-dimensional repositioning to correct maxillary deformities. This procedure is frequently paired with condylectomy or sagittal split ramus osteotomy (SSRO) in patients with hemimandibular hyperplasia (HH) and osteochondroma to comprehensively address both maxillary and mandibular asymmetries. In cases with notable facial asymmetry, mandibular surgeries such as SSRO or condylectomy are essential to complement the correction of maxillary cant and asymmetry, ultimately achieving balanced and harmonious esthetic outcomes.

Xia et al. (2023) examined the outcomes of combining LeFort I osteotomy with SSRO and condylectomy in patients with mandibular osteochondroma. Their study emphasized the importance of precise repositioning of both the maxilla and mandible to ensure effective correction of asymmetry. The combination of these techniques resulted in significant improvements in facial symmetry and occlusal plane leveling, underscoring the value of this multi-procedural approach in achieving stable, long-term outcomes for complex cases of facial asymmetry [39].

##### Outcomes and Long-Term Stability

LeFort I osteotomy provides durable functional and esthetic outcomes for patients with HH and osteochondroma. Correcting occlusal plane canting and restoring facial symmetry significantly enhance masticatory function and overall quality of life. Long-term follow-up studies have reported stable results with minimal complications.

Luo et al. (2017) reported favorable outcomes in patients who underwent LeFort I osteotomy in conjunction with SSRO and condylectomy, noting substantial improvements in TMJ function and facial symmetry without significant postoperative complications [40]. Loureiro et al. (2022) similarly emphasized the utility of postoperative CT imaging in confirming the stable positioning of the maxilla and mandible after LeFort I osteotomy, with no major postoperative complications observed, further supporting the reliability of this combined approach [41].

##### Complications

While LeFort I osteotomy is generally a safe and effective procedure, potential complications, such as neurosensory disturbances, bleeding, and infection, may still occur. These risks can often be minimized with meticulous surgical planning and the integration of advanced techniques like piezosurgery, which allows for precise bone cutting while minimizing impact on surrounding soft tissues. Eltayeb and Ahmad (2017) highlighted the advantages of using piezosurgery during LeFort I osteotomy, specifically in preserving neurosensory function and reducing the risk of nerve injury throughout the procedure [42] (Table 2).

Orthognathic surgery, especially when combined with condylectomy, remains a critical approach in addressing the functional and esthetic deformities associated with hemimandibular hyperplasia (HH) and osteochondroma. The use of advanced technologies, such as 3D surgical planning and virtual guides, has optimized outcomes by enhancing procedural predictability and improving long-term success. Despite potential risks of complications, the overall outcomes of LeFort I osteotomy and related procedures are favorable, offering patients stable and lasting improvements in both function and appearance.

#### 3.3.3. Distraction Osteogenesis

Distraction osteogenesis (DO) is a valuable surgical technique employed to correct significant mandibular deformities and deficiencies, including those resulting from hemimandibular hyperplasia (HH) associated with osteochondroma. DO enables both bone regeneration and soft tissue expansion by gradually stretching the bone, allowing for effective asymmetry correction while reducing the necessity for bone grafting. This approach is especially beneficial in complex cases where traditional reconstructive surgery may be inadequate or less favorable due to the extent of the deformity or the patient’s specific needs.

##### Surgical Technique and Application

Distraction osteogenesis (DO) involves the gradual elongation of bone through controlled mechanical distraction, typically using either external or internal devices. The procedure starts with an osteotomy to separate the bone, followed by a latency period during which the soft tissues and blood supply adjust before mechanical distraction begins. The bone segments are then gradually moved apart at a controlled rate, typically around 1 mm per day, allowing new bone to form in the expanding gap. Once the target length is achieved, a consolidation phase allows the newly formed bone to ossify and harden, ensuring structural stability.

Wang et al. (2000) applied DO to six patients with mandibular defects resulting from tumor resections, reporting successful mandibular lengthening and functional restoration in five of the six cases, illustrating DO’s effectiveness in reconstructive cases [43]. Cheung and Lo (2007) focused on using DO to correct mandibular asymmetry in HH, demonstrating substantial improvements in mandibular length and symmetry, reinforcing DO as a viable approach for complex asymmetry cases [44].

This description of the DO technique and application provides a concise yet comprehensive overview of its methodology and specific outcomes in HH-associated cases, making it suitable for publication. Let me know if you would like to add more studies or details on DO device types and protocols.

##### Outcomes and Long-Term Results

Distraction osteogenesis (DO) has proven highly beneficial for patients with severe mandibular deformities. McCarthy et al. (1992) were pioneers in applying DO for mandibular lengthening, achieving significant improvements in both symmetry and mandibular function across all patients in their study [45]. More recent studies, such as Ow et al. (2010), have reaffirmed DO’s effectiveness, showing that it can successfully correct complex mandibular deformities with favorable long-term outcomes [46].

However, DO is associated with certain complications, primarily device-related issues, which can include infection, mechanical failures, and patient discomfort during the distraction phase. Both Schmitter et al. (2006) and Hoffmann et al. (2014) reported that while DO is effective in correcting mandibular asymmetry and enhancing mandibular length, device-related complications sometimes necessitated secondary procedures to address these issues [47,48] (Table 3).

Distraction osteogenesis remains a valuable technique for addressing severe mandibular deformities, including those associated with HH and osteochondroma. Its ability to enhance symmetry and function in cases where conventional surgery may be insufficient underscores its importance. However, the risk of complications related to the distraction device emphasizes the need for close follow-up and patient management throughout the treatment process.

#### 3.3.4. Total Joint Replacement (TJR)

Total joint replacement (TJR) is a key treatment approach for severe temporomandibular joint (TMJ) pathologies, particularly in cases of hemimandibular hyperplasia (HH) associated with osteochondroma. TJR is generally recommended when other surgical treatments, such as condylectomy, are inadequate, or when there is extensive joint destruction due to pathology. The procedure involves excising the diseased condyle and replacing it with a prosthetic joint, which can be either stock or custom-made to suit the patient’s anatomical and functional requirements.

##### Indications and Prosthetic Options

TJR is indicated for patients with advanced joint disease, recurrent TMJ ankylosis, or extensive osteochondroma affecting the TMJ. It is also a preferred option when joint preservation is not feasible through less invasive surgeries. Prosthetic options include stock and custom-made prostheses, with the choice largely depending on the specific needs of the patient, the extent of disease involvement, and the desired functional and aesthetic outcomes.

Morey–Mas et al. (2011) reported the successful use of a stock prosthesis in a patient who underwent condylectomy for osteochondroma, resulting in excellent functional and aesthetic outcomes post-surgery [25]. Park et al. (2015) similarly found positive results with stock TMJ prostheses, observing significant improvements in mandibular mobility and pain relief 1 year after surgery [24]. In cases with complex anatomical demands, Wilson et al. (2015) highlighted the advantages of custom joint replacement following tumor resection, achieving substantial corrections in severe facial asymmetry [33].

##### Outcomes and Complications

TJR has demonstrated significant benefits for patients with severe TMJ pathologies, offering substantial improvements in function, pain reduction, and facial symmetry. Long-term outcomes are generally favorable, with most patients achieving stable results and improved quality of life. However, complications such as prosthesis loosening, infection, and nerve injury can occur, particularly in complex cases requiring extensive reconstruction.

Loureiro et al. (2022) evaluated postoperative CT scans to monitor prosthesis stability in TJR patients, emphasizing the importance of imaging for early detection of complications and ensuring long-term success [41]. Additionally, Gonzalez–Perez et al. (2016) conducted a 3-year prospective study on TJR using two types of prostheses, noting excellent functional outcomes with minimal complications, although a few patients required minor adjustments for optimal fit [49].

In summary, TJR provides an effective solution for advanced TMJ conditions associated with HH and osteochondroma, especially in cases where other surgical options fall short. The choice between stock and custom prostheses allows for tailored treatment, ensuring optimal functional and aesthetic results. Nonetheless, vigilant follow-up is essential to monitor for potential complications and maintain the longevity of the prosthesis.

##### Complications

While total joint replacement (TJR) generally yields favorable outcomes, complications such as infection, prosthesis loosening, and heterotopic bone formation can occur. A systematic review by Bach et al. (2022) reported a slightly higher complication rate in custom-made prostheses compared to stock prostheses, which may be due to their use in more complex cases. Nevertheless, the overall revision rate for TJR remains low, with most patients experiencing long-term benefits from the surgery [50] (Table 4).

Total joint replacement remains an effective option for managing advanced TMJ pathology, especially in cases involving osteochondroma. Both stock and custom prostheses yield significant improvements in function, mobility, and pain relief. While complications, such as infection or prosthesis failure, can occur, they are relatively rare, and the overall success rate of TJR is high. Long-term follow-up is essential to monitor for potential complications and to ensure the durability of the prosthesis, with most patients experiencing substantial functional and aesthetic benefits over time.

#### 3.3.5. Genioplasty in the Treatment of Hemimandibular Hyperplasia Associated with Osteochondroma

Genioplasty is a valuable adjunctive procedure for managing facial asymmetry caused by hemimandibular hyperplasia (HH) and osteochondroma. This surgical technique focuses on repositioning and reshaping the chin to enhance facial harmony, especially for patients with significant mandibular asymmetry. Genioplasty is frequently combined with other orthognathic surgeries, such as LeFort I osteotomy or sagittal split ramus osteotomy (SSRO), to provide comprehensive correction of facial deformities.

##### Indications for Genioplasty

Genioplasty is indicated in patients with asymmetrical or disproportionate chin positioning due to mandibular overgrowth. It is particularly beneficial when both the lower facial third and the occlusal plane are affected, requiring a holistic approach to address aesthetic and functional concerns.

Rahpeyma and Khajehahmadi (2014) demonstrated that genioplasty, combined with orthognathic surgery, effectively corrected asymmetries due to HH, resulting in significant aesthetic improvements in chin alignment and overall facial symmetry [53]. Genioplasty plays an essential role in aligning the chin with the corrected mandible, ensuring balanced facial proportions in patients with HH.

##### Techniques and Modifications

Sliding genioplasty is the most utilized technique, allowing for precise repositioning of the bony chin segment to align with the newly corrected mandible. Advances in virtual surgical planning and 3D templates have further enhanced the accuracy and predictability of genioplasty outcomes, reducing intraoperative errors.

Li et al. (2016) reported on the use of virtual planning in genioplasty, which led to excellent chin symmetry and predictable outcomes in patients with mandibular deformities secondary to osteochondroma [54]. The precision enabled by these advanced techniques contributes to reliable postoperative results and minimizes the likelihood of asymmetric recurrence.

##### Outcomes and Long-Term Stability

Genioplasty, when integrated with other orthognathic procedures, significantly enhances both functional and aesthetic outcomes. Correcting chin asymmetry improves overall facial harmony, and with proper planning and execution, long-term stability is typically achieved.

Shi et al. (2014) reported successful outcomes with genioplasty in patients with osteochondroma, highlighting substantial improvements in facial balance and function without asymmetry recurrence during long-term follow-up [55]. These findings underscore the critical role of genioplasty in achieving a well-balanced lower facial profile, which is crucial for managing complex craniofacial conditions like HH.

##### Complications and Considerations

Genioplasty is generally associated with minimal complications. However, potential risks include neurosensory disturbances, such as neuropraxia, which may present as hyperesthesia or hypoesthesia. Other possible complications include infection and minor asymmetry recurrence. The application of advanced techniques like piezosurgery, which allows for precise bone cutting, can mitigate these risks by reducing the likelihood of nerve injury.

Rahpeyma and Khajehahmadi (2014) highlighted the importance of nerve repositioning during orthognathic surgery and genioplasty, emphasizing its role in preventing postoperative sensory deficits and facilitating a smoother recovery [53]. Effective nerve management and meticulous surgical technique are crucial to minimizing postoperative complications and achieving optimal outcomes (Table 5).

Genioplasty serves as an essential adjunct in managing HH associated with osteochondroma by enhancing facial symmetry and correcting chin positioning. When combined with orthognathic surgery, it contributes substantially to comprehensive treatment outcomes. Advanced techniques, including 3D surgical planning and nerve repositioning, have improved the precision and predictability of genioplasty, leading to long-term stability with minimal complications. With careful planning and surgical execution, genioplasty offers lasting esthetic and functional benefits for patients with HH.

### 3.4. Synthesis of Results

Managing hemimandibular hyperplasia (HH) associated with osteochondroma necessitates a multidisciplinary surgical approach to address the complex functional and esthetic challenges presented by the condition. The primary goals are to remove pathological growth—due to the neoplastic nature of osteochondroma—and to restore facial symmetry, occlusion, and normal temporomandibular joint (TMJ) function. This synthesis evaluates the effectiveness, outcomes, and potential complications of the reviewed surgical interventions, including condylectomy, orthognathic surgery (LeFort I osteotomy and SSRO), distraction osteogenesis, and total joint replacement (TJR).

#### 3.4.1. Condylectomy

Condylectomy is a foundational technique in treating hemimandibular hyperplasia (HH) associated with osteochondroma, effectively removing pathological condylar growth and improving both facial symmetry and function. This procedure includes high- and low-condylectomy approaches, each suited to specific anatomical and pathological needs.

High Condylectomy: High condylectomy allows for precise removal of extensive lesions. Si et al. (2023) illustrated its precision using 3D-printed cutting guides, which led to significant improvements in occlusion and facial esthetics, with a notably low recurrence rate [23]. Furthermore, Park et al. (2015) demonstrated that combining high condylectomy with bimaxillary orthognathic surgery can address severe asymmetry and occlusal disturbances effectively, showing no recurrence and yielding favorable esthetic and functional outcomes, particularly in cases with extensive condylar involvement [24].

Low Condylectomy: For patients with localized osteochondroma, low condylectomy serves as a conservative, effective option. Morey–Mas et al. (2011) reported favorable long-term stability with positive functional and esthetic results, finding this technique beneficial for patients not requiring complete condylar removal, thus allowing for faster recovery and reducing the need for joint reconstruction [25].

The choice between high and low condylectomy should be guided by the extent of condylar involvement, as well as patient-specific anatomical and functional requirements. High condylectomy is generally more appropriate for extensive lesions requiring thorough resection, while low condylectomy is a reliable approach for smaller, localized growths, offering long-term results with minimal complications.

#### 3.4.2. Orthognathic Surgery (LeFort I Osteotomy and SSRO)

Orthognathic surgery, particularly LeFort I osteotomy and sagittal split ramus osteotomy (SSRO), is essential in addressing the complex facial deformities seen in HH. Combining these orthognathic surgeries with condylectomy has consistently demonstrated significant improvements in facial symmetry, occlusion, and overall functional outcomes.

Studies by Park et al. (2015) and Wilson et al. (2015) found that bimaxillary surgery combined with condylectomy resulted in substantial esthetic and functional enhancements, with stable outcomes maintained over long-term follow-up [24,33]. The integration of virtual surgical planning and 3D templates has further advanced precision and predictability in these procedures. Li et al. (2016) highlighted the effectiveness of using 3D-printed surgical guides in LeFort I osteotomy and SSRO, which led to improved facial symmetry and consistent postoperative results [54]. While Wu et al. (2018) noted a risk of minor occlusal relapse, orthognathic surgery generally provides durable benefits in both function and esthetics [36].

#### 3.4.3. Distraction Osteogenesis

Distraction osteogenesis (DO) is a valuable technique, particularly in cases requiring significant mandibular lengthening where traditional reconstructive options may fall short. DO gradually expands bone and surrounding soft tissue, promoting natural tissue regeneration without the need for grafts.

Ow et al. (2010) demonstrated that DO effectively restored symmetry in patients with substantial mandibular deformities due to HH, achieving durable improvements in both appearance and function [46]. Despite its advantages, DO carries risks such as device malfunction and infection. Studies by Schmitter et al. (2006) and Warren et al. (2001) underscore the importance of close postoperative management to address these potential complications, ensuring optimal outcomes [47,56]. Overall, DO remains a reliable technique for achieving facial symmetry and functional restoration in complex HH cases.

#### 3.4.4. Total Joint Replacement (TJR)

Total joint replacement (TJR) is typically reserved for severe cases of HH associated with osteochondroma, particularly when extensive temporomandibular joint (TMJ) damage precludes less invasive approaches. TJR involves resecting the affected condyle and replacing it with a prosthesis, which can be either stock or custom-made.

Morey–Mas et al. (2011) reported significant functional and esthetic improvements using a stock prosthesis following condylectomy for osteochondroma, supporting TJR’s effectiveness in complex cases [25]. Custom prostheses offer an added degree of precision, as described by Wilson et al. (2015), who used a custom joint replacement to achieve notable facial symmetry corrections in a challenging case, underscoring the benefits of tailored solutions [33]. Long-term studies, such as those by Gonzalez–Perez et al. (2023), confirm the stability and reliability of TJR, particularly with custom prostheses in advanced pathology [51].

Both stock and custom prostheses have shown considerable success in restoring function, improving mobility, and reducing pain, with minimal complications. However, careful postoperative follow-up is essential to monitor prosthesis stability and address any emerging issues, ensuring lasting results.

#### 3.4.5. Genioplasty

Genioplasty serves as an essential adjunctive procedure for patients with HH-related chin deformities, enhancing facial harmony by repositioning and reshaping the chin. This technique is particularly beneficial for patients undergoing orthognathic surgery, as it contributes to comprehensive correction of asymmetry.

Rahpeyma and Khajehahmadi (2014) demonstrated that genioplasty, combined with orthognathic surgery, effectively corrected chin asymmetry, significantly enhancing facial balance in patients with HH [53]. Advances in virtual surgical planning, as reported by Li et al. (2016), have further refined the accuracy and predictability of genioplasty, minimizing postoperative complications and achieving long-term stability in both function and esthetics [54]. By aligning the chin with the newly corrected mandible, genioplasty helps achieve harmonious facial proportions, providing lasting esthetic and functional benefits.

#### 3.4.6. Comparative Outcomes and Overall Stability

The surgical techniques reviewed—condylectomy, orthognathic surgery, distraction osteogenesis, TJR, and genioplasty—each offer unique benefits in treating HH deformities associated with osteochondroma. High and low condylectomy reliably achieve condylar resection, with low recurrence rates and favorable outcomes in symmetry and function. Orthognathic surgery, especially when combined with condylectomy, has been shown to yield significant esthetic and functional results with stable, long-term outcomes. DO provides a valuable alternative for bone lengthening and regeneration, though device-related complications can occur.

Total joint replacement is considered a definitive solution for cases with severe TMJ involvement, while genioplasty offers precise refinements in chin alignment, further enhancing overall facial balance. The integration of advanced technologies, such as 3D printing and virtual surgical planning, has improved the predictability of these techniques, yielding durable results. Regular postoperative follow-up is essential to monitor for potential complications and ensure long-term success, as some cases may experience issues such as infection, prosthesis failure, or minor occlusal relapse.

#### 3.4.7. Conclusions

This synthesis highlights the efficacy of various surgical approaches in managing HH associated with osteochondroma. Each technique presents distinct advantages, and the integration of advanced surgical planning and individualized treatment has significantly enhanced patient outcomes. Although long-term follow-up and attentive postoperative care remain crucial for maintaining stability and function, advancements in personalized prosthetics and digital planning offer promising avenues for further improvements. Collectively, these interventions contribute to both functional restoration and esthetic improvement, underscoring the importance of a tailored, multidisciplinary approach for optimal patient care.

## 4. Discussion

### 4.1. General Interpretation of Results in the Context of Other Evidence

This systematic review confirms the efficacy of various surgical approaches for managing hemimandibular hyperplasia (HH) associated with osteochondroma, aligning with outcomes reported in other studies. Procedures such as condylectomy (high and low), orthognathic surgery (LeFort I osteotomy and sagittal split ramus osteotomy), distraction osteogenesis (DO), total joint replacement (TJR), and genioplasty consistently demonstrated success in enhancing facial symmetry, occlusion, and temporomandibular joint (TMJ) function.

The results support prior findings emphasizing the importance of removing the pathological condyle to achieve esthetic and functional balance. High condylectomy effectively reduced recurrence risks, while low condylectomy offered faster recovery without necessitating prosthetic replacement, highlighting its value for localized lesions [11,16]. These findings align with the literature that advocates condylectomy as a reliable method for managing condylar osteochondroma while preserving TMJ function [10,26].

Orthognathic surgery, particularly when combined with condylectomy, emerged as a primary approach for achieving both functional and esthetic correction in HH. Studies, including those by Park et al. (2015) and Wilson et al. (2015), reported stable, long-term outcomes with combined bimaxillary surgery and condylectomy, underscoring the benefits of such combined interventions [24,33]. The integration of virtual surgical planning and 3D-printed guides has further improved the accuracy of orthognathic procedures, reflecting a broader trend toward using advanced tools that yield more predictable, stable outcomes [23,35].

DO offers unique advantages by enabling simultaneous bone lengthening and soft tissue expansion. Consistent with previous research, this review highlights DO’s effectiveness in addressing severe mandibular deformities, with successful functional correction frequently reported [44]. However, device-related complications, such as infection and mechanical failure noted by Schmitter et al. (2006) and Warren et al. (2001), indicate a need for careful management to optimize outcomes [47,56].

TJR remains an important option for cases with extensive TMJ damage or recurrence after prior surgeries. This review supports existing research demonstrating the effectiveness of both stock and custom prostheses for restoring joint function and symmetry, with custom prostheses providing enhanced precision in complex cases [49]. TJR has shown consistent improvements in function, mobility, and pain relief, with minimal complications, reinforcing its reliability for advanced TMJ reconstruction [50,51].

Finally, genioplasty serves a critical role as an adjunctive procedure, especially for patients with pronounced chin asymmetry. By reshaping and repositioning the chin, genioplasty enhances facial harmony, particularly when combined with orthognathic surgery. Studies by Rahpeyma and Khajehahmadi (2014) highlight the procedure’s effectiveness in restoring chin symmetry, and 3D surgical planning has further improved its precision and predictability [53,54].

In conclusion, this review affirms that a multidisciplinary approach—integrating condylectomy, orthognathic surgery, DO, TJR, and genioplasty—effectively addresses the anatomical and functional challenges of HH associated with osteochondroma. The application of advanced surgical planning and individualized treatment strategies has refined outcomes, indicating an evolving standard of care that promises increased precision and durable results for patients with complex craniofacial deformities.

### 4.2. Limitations of the Evidence Included in the Review

While the studies reviewed indicate favorable outcomes for managing hemimandibular hyperplasia (HH) associated with osteochondroma, several limitations must be noted. A significant portion of the studies comprises case reports or case series, which are inherently limited by small sample sizes and lack of control groups. Without randomized controlled trials (RCTs) in this field, the strength of evidence is limited, making it challenging to generalize findings across broader patient populations [26,27,30].

Additionally, many studies did not consistently report postoperative complications, limiting a comprehensive assessment of the true risk profile associated with each surgical technique. The shortage of long-term follow-up data in some studies further complicates evaluations of surgical stability, as recurrence rates and functional outcomes may not be fully captured. Variation in follow-up durations also poses challenges in comparing techniques, particularly regarding recurrence and sustained functional improvements over time [47,56].

This review highlights the need for larger, prospective studies with standardized follow-up protocols to strengthen the evidence base, providing clearer guidance for clinical decision-making in treating HH associated with osteochondroma.

### 4.3. Limitations of the Review Process Used (23c)

Despite the rigorous selection criteria applied in this systematic review, certain limitations of the review process should be acknowledged. The inclusion of only English-language studies may have introduced language bias, potentially excluding relevant research published in other languages. Although a comprehensive search across multiple databases was performed to mitigate selection bias, it is possible that some studies meeting the inclusion criteria were not identified. This limitation could lead to an incomplete representation of the available evidence, particularly if excluded studies reported differing outcomes or complication rates, which may impact the overall conclusions drawn from this review [36].

Another limitation stems from the variability in surgical techniques across the studies included. Differences in surgical protocols, patient selection criteria, and postoperative care present challenges in standardizing comparisons and assessing the overall effectiveness of each technique. This variability in methods and outcomes complicates the interpretation of results, as the findings may be affected by unique aspects of individual studies. Consequently, while this review provides insights into the surgical management of hemimandibular hyperplasia (HH) associated with osteochondroma, the conclusions should be interpreted with caution. Further research, particularly with standardized protocols and larger, controlled studies, is necessary to establish more definitive guidelines for effectively managing this condition [26,30].

### 4.4. Implications for Practice, Policy, and Future Research

This systematic review underscores the effectiveness of multiple surgical techniques in managing hemimandibular hyperplasia (HH) associated with osteochondroma, with important implications for clinical practice, policy, and future research:

- **Personalized Treatment Plans**: Surgeons should develop tailored surgical interventions based on patient-specific factors, including the extent of condylar involvement and presence of temporomandibular disorders. Both high- and low-condylectomy approaches provide reliable outcomes. However, selecting the most suitable approach requires careful consideration of each patient’s unique anatomical and functional needs [10,11].
- **Advances in 3D Surgical Planning**: The integration of virtual surgical planning and 3D-printed guides has significantly enhanced the precision of procedures, particularly for orthognathic surgeries and genioplasty. Incorporating these technologies into standard clinical practice can improve surgical accuracy, reduce complications, and support more predictable outcomes, reinforcing the value of technology-driven surgical planning [12,23].
- **Postoperative Monitoring**: Due to the potential risk of occlusal relapse, especially after orthognathic surgery, establishing robust, long-term follow-up protocols is essential. Surgeons are encouraged to adopt comprehensive postoperative monitoring to promptly detect and address issues, ultimately improving long-term patient outcomes and satisfaction [34,36].
- **Future Research Directions**: There is a need for randomized controlled trials to strengthen the evidence base for these surgical interventions. Future studies should aim to standardize surgical protocols and postoperative care to allow for more accurate comparisons between techniques. Moreover, long-term follow-up studies are crucial for evaluating the durability of surgical outcomes and assessing recurrence risks, which is vital for ensuring sustained improvements in patient care [26,29].

In conclusion, this review provides valuable insights into the management of HH associated with osteochondroma. While the reviewed surgical techniques generally show effectiveness, continued advancements in surgical planning technologies and high-quality research are essential to refine these procedures further and achieve lasting, stable outcomes. Through ongoing research and innovation, the field can enhance treatment efficacy and optimize the quality of life for patients with HH.

## 5. Conclusions

This systematic review evaluated the effectiveness of various surgical approaches for managing hemimandibular hyperplasia (HH) associated with osteochondroma, specifically focusing on condylectomy, orthognathic surgery, distraction osteogenesis, total joint replacement (TJR), and genioplasty. Each of these approaches has shown favorable outcomes in restoring facial symmetry, correcting occlusion, and enhancing mandibular function.

Condylectomy, both high and low, remains a cornerstone procedure that consistently yields positive results, particularly when combined with orthognathic surgery. This combination significantly enhances facial symmetry and occlusal function, proving especially beneficial in complex cases.

Orthognathic surgery techniques, including LeFort I osteotomy and sagittal split ramus osteotomy (SSRO), play a crucial role in addressing craniofacial deformities. These procedures are essential for achieving balanced occlusion and facial symmetry, underscoring their value in comprehensive HH management.

Distraction osteogenesis (DO) is a valuable option for patients requiring mandibular lengthening, especially when traditional grafting methods are inadequate. While DO offers substantial benefits, long-term stability hinges on meticulous management of potential complications, such as device malfunction or infection.

Total joint replacement (TJR) offers a definitive solution for cases with severe temporomandibular joint (TMJ) involvement, particularly when previous surgeries have failed or when osteochondroma has extensively compromised joint function. Both stock and custom prostheses have been shown to significantly enhance joint mobility, reduce pain, and improve quality of life in these patients.

Genioplasty is a vital adjunctive procedure, particularly in achieving facial balance by correcting chin asymmetry associated with HH. Advanced surgical techniques, such as virtual planning and 3D printing, have greatly improved the precision of genioplasty and other procedures, contributing to more predictable and esthetic outcomes.

Despite the generally low risk of bias among the studies included, limitations related to small sample sizes and limited follow-up periods remain. Future research should prioritize larger cohort studies with extended follow-up to provide more definitive data on long-term outcomes and potential complications. Technological advancements, especially in virtual surgical planning and 3D printing, continue to enhance surgical precision and patient-specific care.

In conclusion, the surgical approaches reviewed here are effective for achieving desired functional and esthetic outcomes in patients with HH associated with osteochondroma. Individualized treatment plans, tailored to each patient’s unique anatomical and functional needs, are essential. Long-term follow-up is critical for monitoring complications and ensuring stable results. Ongoing advancements in surgical techniques and technologies will further refine these approaches, enhancing patient care and outcomes in the management of this complex condition.

## Figures and Tables

**Table 1:** High- and low-condylectomy outcomes for hemimandibular hyperplasia associated with osteochondroma.

Authors	Study Design	Sample Size	Surgical Technique(s)	Outcomes Assessed	Follow-Up Duration	Key Results	Complications
Si et al. (2023) [23]	Comparative study	21	High condylectomy (3D-printed guide)	Resection precision, recurrence	12 months	Precise resection, no recurrence	None
Mehra et al. (2016) [30]	Comparative study	24	Low condylectomy vs. complete condylectomy	Joint function, recovery	12–36 months	Faster recovery with low condylectomy, similar outcomes to complete condylectomy	Minimal recurrence risk
Park et al. (2015) [24]	Case report	1	High condylectomy + orthognathic surgery	Symmetry, occlusion	12 months	Significant improvement, no recurrence	None
Mamatha et al. (2015) [27]	Case report	1	Low condylectomy	Facial symmetry, joint function	12 months	Successful recovery, no recurrence	None
Kim et al. (2015) [28]	Case series	5	Low condylectomy	Symmetry, joint function	12–24 months	Restored symmetry, no recurrence	Minimal complications
Wolford et al. (2014) [10]	Retrospective review	37	Low condylectomy + orthognathic surgery	Functional outcomes, esthetics	24 months	Functional and esthetic improvements, minimal complications	None
Morey–Mas et al. (2011) [25]	Case report	1	Low condylectomy + prosthesis	Joint function, occlusion	24 months	Stable occlusion, no recurrence	None

**Table 2:** Outcomes of orthognathic surgery and LeFort osteotomy for hemimandibular hyperplasia associated with osteochondroma.

Authors	Study Design	Sample Size	Surgical Techniques(s)	Outcomes Assessed	Follow-Up Duration	Key Results	Complications
Qi et al. (2021) [31]	Prospective study	20	LeFort I + SSRO	Skeletal symmetry, occlusionn	24 months	Significant improvement, stable outcomes	None
Wu et al. (2018) [36]	Retrospective review	10	SSRO + condylectomy	Occlusal function, recurrence	24 months	Stable outcomes, no recurrence	None
Luo et al. (2017) [40]	Case report	1	SSRO + TMJ reconstruction	TMJ function, occlusion	12 months	Successful restoration, no complications	None
Park et al. (2015) [24]	Case report	1	LeFort I + condylectomy	Occlusion, facial symmetry	12 Months	Significant improvements in TMJ function, no recurrence	None
Wilson et al. (2015) [33]	Case series	15	SSRO + condylectomy	Occlusal function, symmetry	24 months	Improved function, stable esthetics	None
Lim et al. (2014) [34]	Case series	12	LeFort I + SSRO	Facial symmetry, occlusion	36 months	Stable outcomes, no recurrence	None

**Table 3:** Distraction osteogenesis outcomes.

Authors	Study Design	Sample Size	Surgical Technique(s)	Outcomes Assessed	Follow-Up Duration	Key Results	Complications
Hoffmann et al. (2014) [48]	Case series	8	DO for mandibular lengthening	Mandibular length, function	24 months	Improved length, some mechanical failures	Device-related complications
Ow et al. (2010) [46]	Retrospective study	15	DO for mandibular deformities	Symmetry, functional improvement	24 months	Favorable long-term outcomes	None
Cheung and Lo (2007) [44]	Case series	12	DO for hemimandibular hyperplasia	Mandibular length, symmetry	18 months	Significant improvements in symmetry and length	None
Schmitter et al. (2006) [47]	Retrospective review	9	DO + MRI assessment	Mandibular condyle, occlusion	36 months	Long-term improvements, occasional secondary procedures	Device-related issues in a few cases
Wang et al. (2000) [43]	Case series	6	DO for tumor resection defects	Occlusion, mandibular function	24 months	Successful lengthening in 5 cases, restored function	None
McCarthy et al. (1992) [45]	Prospective study	10	DO for mandibular lengthening	Mandibular symmetry, function	36 months	Improved symmetry and function	None

**Table 4:** Total joint replacement outcomes.

Authors	Study Design	Sample Size	Surgical Technique(s)	Outcomes Assessed	Follow-Up Duration	Key Results	Complications
Gonzalez–Perez et al. (2023) [51]	Case series	21	TJR post-TMJ tumor resection	Mobility, pain	36 months	Substantial improvements in mobility, pain relief	Minimal complications
Bach et al. (2022) [50]	Systematic review	27 studies	Stock vs. custom TJR	Prosthesis failure, infection	Varies	Slightly higher complication rate with custom prostheses	Prosthesis loosening, infection
Gonzalez–Perez et al. (2016) [49]	Prospective study	15	Comparison of stock vs. custom prostheses	Function, stability	36 months	No significant difference in stability	4% required revision
Wilson et al. (2015) [33]	Case report	1	Custom joint replacement	Facial asymmetry correction	18 months	Complete correction of facial asymmetry	None
Lee et al. (2013) [52]	Case series	2	TJR with stock prosthesis	Mandibular mobility, pain	12 months	Improved mobility and pain relief	None
Morey–Mas et al. (2011) [25]	Case report	1	TJR with stock prosthesis	Function, aesthetics	24 months	Excellent functional and aesthetic results	None

**Table 5:** Genioplasty outcomes in hemimandibular hyperplasia.

Authors	Study Design	Sample Size	Surgical Techniques(s)	Outcomes Assessed	Follow-Up Duration	Key Results	Complications
Li et al. (2016) [54]	Case series	10	Genioplasty + virtual surgical planning	Chin projection, facial symmetry	24 months	Significant esthetic improvement, excellent outcomes	None
Rahpeyma and Khajehahmadi (2014) [53]	Case series	5	Genioplasty + orthognathic surgery	Nerve repositioning, facial symmetry	18 months	Significant improvement in symmetry, minimal nerve damage	None
Shi et al. (2014) [55]	Case series	12	Genioplasty + one-stage condylectomy	Chin symmetry, occlusion	24 months	Improved facial balance and stable occlusion	None

## Supplementary Materials

The following supporting information can be downloaded at: https://www.mdpi.com/article/10.3390/jcm13226988/s1, PRISMA 2020 Checklist.
